# A case in which a capnometer was useful for diagnosing laryngospasm following administration of sugammadex

**DOI:** 10.1186/s40981-017-0111-8

**Published:** 2017-08-15

**Authors:** Keito Kou, Takeshi Omae, Saiko Wakabayashi, Sonoko Sakuraba

**Affiliations:** Department of Anesthesiology and Pain Clinic, Juntendo University Shizuoka hospital, 1129 Izunokuni, Shizuoka, Japan

**Keywords:** Capnometer, Rocuronium, Sugammadex, Continuous positive airway pressure, Bronchospasm

## Abstract

**Background:**

Sugammadex has been reported to cause upper-airway obstruction, such as laryngospasm or bronchospasm. These two conditions are treated using different approaches, but the differential diagnosis is difficult.

**Case presentation:**

We describe a case in which general anesthesia was administered via endotracheal intubation, in combination with brachial-plexus block, for arthroscopic surgical treatment of a rotator-cuff tear caused by recurrent shoulder dislocation. The total dose of rocuronium administered was 90 mg, and the last dose of 10 mg was given 15 min before the end of the surgery. Sugammadex was intravenously administered at 100 mg to reverse the effect of rocuronium after the operation ended. After extubation in this case, we placed a mask firmly around the patient’s mouth, and thus, there was no air leakage around the mask. We detected upper-airway obstruction that was presumably attributable to administration of sugammadex. The end-tidal carbon dioxide (EtCO_2_) concentration was undetectable on a capnometer. Although 100% oxygen was administered at 10 L/min via a facemask, oxygen saturation (SpO_2_) decreased to approximately 70%. With suspected onset of laryngospasm, continuous positive airway pressure with 100% oxygen at 10 L/min was started at 30 cm H_2_O. The patient’s airway obstruction resolved after a short time.

**Conclusion:**

The use of a capnometer facilitated the diagnosis of laryngospasm and allowed us to administer appropriate treatment after administration of sugammadex.

## Background

Sugammadex, an antagonist of muscle relaxants, has been reported to cause upper airway obstruction, such as laryngospasm [1–3] or bronchospasm [3, 4]. We herein report our experience with a case in which a capnometer was useful for the differential diagnosis of upper airway obstruction following administration of sugammadex.

## Case presentation

**Fig. 1:**
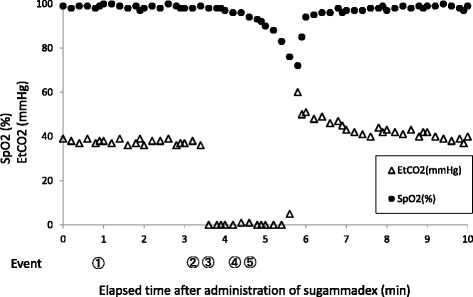
Changes in end-tidal carbon dioxide and arterial oxygen saturation of pulse oxymetry after sugammadex administration. Event: ① intravenous injection of sugammadex, ② extubation, ③ disappearance of end-tidal carbon dioxide (EtCO2), ④ decline of arterial oxygen saturation, ⑤ start of continuous positive airway pressure

A 25-year-old man with a height of 168 cm and weight of 68 kg underwent arthroscopic surgery to treat a rotator cuff tear caused by recurrent shoulder dislocation. He had no remarkable medical history. General anesthesia was induced with propofol (100 mg), remifentanil (0.3 μg/kg/min), and rocuronium (40 mg), while an 8.0-mm endotracheal tube was inserted for airway management. Additionally, a brachial plexus block was administered with 0.25% levobupivacaine (30 mL) through an interscalene approach under ultrasonographic guidance, and an indwelling catheter was placed. While the patient was placed in the beach-chair position during surgery, anesthesia was maintained with oxygen 1 L/min, air 2 L/min, 1.5% sevoflurane, and remifentanil 0.2-0.3 μg/kg/min. The patient was monitored using five-lead electrocardiography, pulse oximetry, and capnometry. The total dose of rocuronium administered was 90 mg, and the last dose of 10 mg was given 15 min before the end of the surgery. The operative time was 3 h and 21 min. Continuous infusion of remifentanil was discontinued simultaneous with the end of surgery. Administration of sevoflurane was discontinued 5 min later. Sugammadex was intravenously administered at 100 mg to reverse rocuronium. Since the patient regained clear consciousness and spontaneous breathing at 10 mL/kg, the intubation tube was removed. Immediately after extubation, he presented with retractive breathing and stridor. According to the capnometer readings, the end-tidal carbon dioxide (EtCO_2_) concentration was undetectable. Although 100% oxygen was administered at 10 L/min via a facemask, oxygen saturation (SpO_2_) decreased to approximately 70%. Onset of laryngospasm was suspected, and thus, 100% oxygen was started at 10 L/min with continuous positive airway pressure (30 cm H_2_O). The patient’s airway was reopened after a while. Consequently, both stridor and retractive breathing resolved. The EtCO_2_ waveforms became detectable with a capnometer, and SpO_2_ also improved to 100% (Fig.1). The patient was monitored in the operating room for 30 min. After a stable level of consciousness and stable respiratory patterns were confirmed, he was discharged from the operating room. No recurrence of laryngospasm was observed throughout the postoperative course.

## Discussion

This patient presented with retractive breathing, stridor, and decreased SpO_2_ immediately after extubation. We suspected that these symptoms were attributable to bronchospasm or laryngospasm associated with administration of sugammadex. It has been reported that sugammadex may cause bronchospasm [3]. This is reportedly more likely in patients with a history of respiratory disease, such as bronchial asthma. Meanwhile, bronchospasm is triggered by various mechanical (e.g., endotracheal intubation), chemical, and pharmacological stimuli. While its intraoperative respiratory adverse event is approximately 1.7%, bronchospasm and laryngospasm are complications in which deaths have been reported in some severe cases [5–7]. The most common pathological condition recognized as bronchospasm is asthma. When considering the treatment for bronchospasm, volatile anesthetic agents are useful because with the exception of desflurane, they have a bronchodilator effect [8]. Desflurane increases airway resistance at a high alveolar concentration [8]. When bronchospasm is severe, it is difficult to use volatile anesthetic agents. Propofol relaxes the airway reflex [9]. Beta-stimulants, such as salbutamol and epinephrine, can also be administered intravenously [10]. Anticholinergic drugs, such as tiotropium bromide, block parasympathetic constriction of bronchial smooth muscle [11]. Intravenous magnesium sulfate is also effective for bronchospasm [12].

Laryngospasm is defined as glottis narrowing caused by reflective contraction of the laryngeal muscles. It is classified into complete and incomplete types. The complete type is referred to as a condition in which the immobility of the back leads to complete failure of ventilation, whereas the incomplete type is referred to as a condition in which the motion of the thorax is accompanied by inspiratory stridor and limited motion of the back. When incomplete laryngospasm progresses to the complete type, inspiratory stridor disappears [13, 14]. Laryngospasm frequently occurs in ambulatory anesthesia [15], while its occurrence has also been reported during orthopedic surgery [16, 17], laparotomy [16], and craniotomy [18]. Laryngospasm is more common in young people than in adults. In fact, its incidence has been reported to reach 14% in those aged less than 6 years [19]. A definitive diagnosis of this condition is made by examination of the vocal cords with a bronchial fiberscope [20]. When laryngospasm is diagnosed, the irritating stimulus by any triggering factor should be removed. Positive-pressure ventilation with 100% oxygen, which forces the chin forward with strong pressure from behind the ascending rami of the jaw, is required for laryngospasm [21]. When this fails, endotracheal intubation is required. Anesthesia should be deepened with an intravenous anesthetic. For endotracheal intubation, succinylcholine is widely used as a muscle relaxant [21]. It has been reported that succinylcholine is administered to avoid significant hypoxia in approximately 25 to 50% of these cases [22]. If it is difficult to intubate using an endotracheal tube, cricothyroidotomy or tracheotomy is needed to access the airway rapidly [23]. Prolonged laryngospasm may cause serious complications, such as cardiac arrest, aspiration, and negative pressure pulmonary edema [23]. Bronchospasm and laryngospasm should be differentiated soon after onset because they differ in terms of treatment strategies and subsequent complications.

In our case, a capnometer was useful for differentiating between bronchospasm and laryngospasm. Although a definitive diagnosis of laryngospasm is made by examination of the vocal cords with a bronchial fiberscope [20], there was insufficient time to perform bronchial fiberscopy in order to prevent progression to NPPE. No waveform appears in capnometer readings of laryngospasm, which causes complete occlusion of the upper respiratory tract; however, they reveal waveform patterns of occlusive respiratory disorders and provide EtCO_2_ values in most cases of bronchospasm. In our case, laryngospasm was diagnosed because EtCO_2_ waveforms were absent on the capnometer readings, and continuous positive airway pressure with 100% oxygen was applied through a facemask, resulting in relief of symptoms.

Because there are reported cases of NPPE that occurred a few hours after extubation [24], adequate follow-up is necessary. However, our patient presented with neither subjective symptoms nor abnormal radiographic findings after surgery.

## Conclusions

After extubation in this case, we detected upper airway obstruction that was presumably attributable to administration of sugammadex. The treatment strategies for upper airway obstruction vary depending on the cause. The use of a capnometer facilitated the diagnosis of laryngospasm and allowed us to administer appropriate treatment.
